# Steps Towards Comprehensive Heat Communication in the Frame of a Heat Health Warning System in Slovenia

**DOI:** 10.3390/ijerph17165829

**Published:** 2020-08-12

**Authors:** Tjaša Pogačar, Zala Žnidaršič, Lučka Kajfež Bogataj, Zalika Črepinšek

**Affiliations:** 1 Centre of Agrometeorology, Department of Agronomy, Biotechnical Faculty, University of Ljubljana, Jamnikarjeva 101, 1000 Ljubljana, Slovenia; lucka.kajfez.bogataj@bf.uni-lj.si (L.K.B.);zalika.crepinsek@bf.uni-lj.si (Z.Č.); 2 Department of Physics, Faculty of Mathematics and Physics, University of Ljubljana, Jadranska 19, 1000 Ljubljana, Slovenia; zala.znidarsic@gmail.com

**Keywords:** heat health warnings, occupational heat stress, stakeholders, meteoalarm, awareness

## Abstract

Occupational heat stress has an important negative impact on the well-being, health and productivity of workers and should; therefore, be recognized as a public health issue in Europe. There is no comprehensive heat health warning system in Slovenia combining public health measures with meteorological forecasts. The aim of this research was to provide insight into the development of such a system in Slovenia, turning the communication from the current meteoalarm into a broader system that has more information for different social groups. To achieve this goal, the following steps were used: Analysis of summer temperatures and issued meteoalarms, a survey of the general knowledge about heat among the public, organization and management of two stakeholder symposia, and a final survey on workers’ opinions on heat stress and measures, supplemented by interviews with employers. Summer average daily temperature distributions in Slovenia changed during the investigated period (1961–2019) and the mean values increased over time by 2–3 °C. Additionally, the number of days with fulfilled yellow (potentially dangerous) and especially orange (dangerous) meteoalarm conditions increased significantly after 1990. The survey of the general public about heat stress and warnings showed that efforts to raise awareness of heat issues need to be intensified and that public health measures should effectively target vulnerable groups. Stakeholder symposia and further surveys have shown that awareness and understanding of the negative effects of heat stress on health and productivity are still quite low, so effective ways of disseminating information to different sectors while striking the best balance between efficiency, feasibility and economic cost have to be found.

## 1. Introduction

In Slovenia, the average summer air temperature is increasing and there is a close correlation between the average summer air temperature and the number of hot days, which is expected to increase [1,2]. The shift of the statistical distribution was unable to explain the record-breaking summer of 2003, so it was shown in 2004 that the European summer climate might experience a pronounced increase in year-to-year variability in response to greenhouse-gas forcing [3]. Europe’s hot summer temperatures [4] are becoming an increasing challenge in various social environments. The population groups most affected by heat waves are older people [5,6], whose body temperature control is impaired due to physiological changes, chronic diseases, the intake of certain drugs and lifestyle, which can quickly lead to dehydration [7]. It is recommended that heat warning thresholds for the general public should be based on temperature—mortality relationships because the efficiency is higher compared to statistical cutoff points (e.g., percentiles of the average temperature distribution) [8,9]. However, it is difficult to overcome the problem of small statistical samples in Slovenia, which has a total of only 2 million inhabitants and; therefore, a very low number of heat-related deaths [7].

In addition, there is not enough attention to the heat exposure of those who work outdoors or near hot equipment [10,11]. The frequent occurrence of various, even very serious, heat stress symptoms and diseases, as well as productivity losses, during or after heat waves has been observed in case studies among Slovenian workers in the manufacturing industry [1,12], agriculture [13] and outdoor work in various sectors [14]. The number of Slovenian workers in the 55–64 age group rose from 57,000 to 109,000 in the period 2008–2018 [15]. With increasing age, workers’ resistance to heat stress decreases, which has further negative effects on health and productivity. It is; therefore, not surprising that more than half (56%) of the estimated total economic cost of the effects of climate change in 2030 is attributed to heat at work [16]. Occupational heat stress has an important impact on the well-being, health and productivity of workers [17,18] and should; therefore, be recognized not only as an occupational but also as a public health issue.

In Slovenia, there is no comprehensive heat health warning system that “would combine public health actions with meteorological forecasts with active identification and care of persons at risk” [19]. Similarly, among the 53 WHO European Region member states, the assessment in 2013 showed that only 18 countries have developed heat health action plans [20]. In 2019, among the 16 EU countries, Slovenia was one of only two countries with heat warnings that are not part of a heat health plan [8]. The meteoalarm issued by the Meteorological Office of the Slovenian Environment Agency is based on the average and maximum daily temperature, with different thresholds used for different levels. It is published on the web five days in advance, with an explanation of the situation. In case of high heat, the meteoalarm is mentioned in the forecast on TV and radio, and the National Institute of Public Health provides advice on the web and social media. Occupational heat stress is not taken into account. Furthermore, it is worrying that the passive dissemination of advice on heat prevention is likely to remain ineffective given the current state of knowledge about high-risk groups [19]. Heat health warning systems should include predictions not only of heat, but also of possible health consequences, triggering effective and timely response plans for vulnerable groups, communication of prevention measures, and evaluation and review of systems [8]. It is important not to raise concern too soon which could lead to a loss of public trust.

This paper aims to provide insight into the development of comprehensive heat communication in the frame of a heat health warning system in Slovenia: from the current meteoalarm to a broader communication with more information for different social groups. Our climatological analysis, research among general public and workers, and stakeholder communication during two symposia aimed to provide useful experiences and best practices on the way towards a heat health warning system in Slovenia, which we assume to be helpful also for other countries. More precisely, we considered four main steps: (1) Preparing comprehensive information on the climatological situation and the issued heat warnings during summer in Slovenia, (2) assessing the general knowledge of the public on heat stress, (3) providing an opportunity for different stakeholders to meet and agree on ideas, and (4) identifying the issues workers and employers face in terms of occupational heat stress.

## 2. Materials and Methods

**Figure 1:**
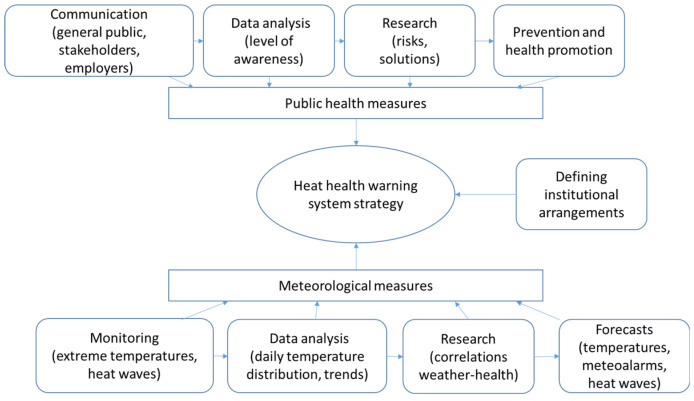
The scheme of a heat health warning system strategy based on the meteorological and public health measures.

On the way towards improved heat warnings and the potential development of a heat health warning system, we applied four steps and described our experiences. We consider it important to cover both meteorological and public health measures in order to create a solid basis for the future development of the warning system (Figure 1). The first step, the climatological analysis, served to obtain more user-friendly and accessible climate information. Although meteorological measures are well covered by the Slovenian Environment Agency (ARSO), the more general approach to link them to public policies is often missing. This was further addressed in the second step, research of general knowledge on heat stress. Both the results and the topics of the meteorological/public health measures were included in two symposia (third step), where the role of institutions, optimal communication channels, possible education measures and the use of the Heat-Shield platform [21] for employers and employees were discussed. The latter was the main reason to include research on occupational heat issues and to involve workers as an important part of the overall heat health warning system.

### 2.1. First Step: Analysis of Summer Temperatures and Issued Meteoalarms

An analysis was conducted to determine the background of the problem of rising summer temperatures, which was used in further communication with stakeholders to describe the escalating problem. The daily air temperatures for June, July and August in the period 1961–2019 were collected from the ARSO archive for six locations in Slovenia: Bilje (lon. 13.624°, lat. 45.895°, 55 m above sea level (a.s.l.)—measurements started in 1962, Ljubljana (lon. 14.512°, lat. 16.065°, 299 m a.s.l.), Celje (lon. 15,255°, lat. 46.236°, 244 m a.s.l.), Murska Sobota (lon. 16.191°, lat. 46.652°, 188 m a.s.l.), Novo mesto (lon. 15.177°, lat. 45.801°, 220 m a.s.l.) and Postojna (lon. 14.193°, lat. 45.766°, 533 m a.s.l.). The distributions of all summer temperatures were plotted for four 30-year periods: 1961–1990, 1971–2000, 1981–2010 and 1990–2019, including the fitted normal distributions obtained using the R software environment [22]. The percentages of summer days with an average daily temperature above three thresholds (22, 24 and 26 °C) were calculated to get a better idea of the ongoing changes.

Since 2008, the Slovenian Meteorological Service of ARSO has been warning of dangerous weather conditions via the web using the European meteoalarm system [23]. In 2011, warnings of extremely high temperatures were included in the ARSO meteoalarm system. The colour scale (Table 1) indicates the degree of weather risk and the possible consequences. The criteria for each warning level (colour) are based on the Slovenian regional climate, assuming that the inhabitants of the different regions are accustomed to their own local conditions. Since there is no archive on the number of meteoalarms issued due to heat, the number of days with fulfilled criteria for each level was calculated for the same six locations as above. Bilje is located in the southwestern region, which has the mildest criterion due to its sub-Mediterranean climate with the hottest summers in Slovenia. The same criterion applies to Novo mesto in the southeastern region, where summer temperatures are also higher than elsewhere. Slightly lower are the criteria for meteoalarms at other places in the central (Ljubljana, Postojna) or northeastern regions (Murska Sobota, Celje), with a subcontinental climate.

### 2.2. Second Step: Research on the General Knowledge about Heat during Summer

The survey was conducted in the summer of 2017 among 213 citizens, in front of five shopping centers in Ljubljana, to get an overview of the general public’s attitude to heat warnings and heat stress. They were asked to participate in the study and gave their oral consent, responding in their own language. Men (63%) and persons under 50 years of age (61%) were in the majority. Their education was very heterogeneous: About one-third had no schooling or only primary school, one-third had secondary school and one-third had completed university. The questionnaire contained questions on knowledge about the national heat warning system, heat stress and heat sensitivity, which were the same as in the published questionnaire by Van Loenhout et al. [25] and Gil Cuesta et al. [26], which allowed for comparison with some other European cities (Brussels and Amsterdam [25], and Lisbon and Madrid [26]). No possible answers were given to the respondents.

### 2.3. Third Step: Organisation and Management of Two Stakeholder Symposia

The two symposia were organized by Slovenian Heat-Shield project partners, the Jozef Stefan Institute and the Biotechnical Faculty of the University of Ljubljana, in June 2018 and June 2019, to bring together different stakeholders concerned with heat stress or heat health warnings and regulations. The basic idea was to present current trends in summer temperatures, climate projections, results of general knowledge research and our previous work (e.g., [1,12]) and to give them the opportunity to present their work or point of view. The participants (20–30 at each meeting) came from the National Institute for Public Health, the Slovenian Environment Agency, the Association of Free Trade Unions of Slovenia, the Biotechnical Faculty and the Faculty of Medicine of the University of Ljubljana, the Clinical Institute of Occupational, Traffic and Sports Medicine, the Agricultural Advisory Service, the Slovenian Traffic Safety Agency, the media, nongovernmental organizations (NGOs) and management representatives of several companies with heat problems. The symposia were designed as a combination of lectures and round tables.

There were four presentations at the first symposium. The first (Biotechnical Faculty, Ljubljana, Slovenia) explained climate change in Slovenia—in particular, the changing characteristics of heat waves. In the second report (ARSO) on the meteoalarm heat warning system in Slovenia, the need for a timely warning system was stressed and uncertainties in forecasting were presented. The third topic (Clinical Institute of Occupational, Traffic and Sports Medicine, Ljubljana, Slovenia) dealt with the consequences of sun and heat exposure. The fourth presentation (Josef Stefan Institute, Ljubljana, Slovenia, and Biotechnical Faculty) was about the case study in the production company with a survey conducted on workers’ perceptions of heat stress, temperature conditions compared to overall productivity and neural networks learning from the measured data tested to predict factory conditions and productivity based on weather forecasts. The goals of the three roundtables were to gather information on heat stress and its mitigation from various sectors and levels (ARSO, institutes, media and companies) and to encourage cooperation between them.

The second symposium started with a presentation on climate trends and projections of different heat indices in Slovenia (ARSO), followed by another on the effects of heat on the human body and thermoregulation in the workplace (Josef Stefan Institute). The next presentation was on the most vulnerable groups due to heat stress (National Institute of Public Health, Ljubljana, Slovenia), on heat exhaustion in the mountains (by a medical doctor and mountain rescuer), and on the effects of heat on drivers. The second part dealt with occupational heat stress. The Heat-Shield platform was presented (Biotechnical Faculty) as a useful heat health warning system for employers and employees. The production company “odelo” gave an example of how they reduce the negative effects of heat, and a representative of the Association of Free Trade Unions of Slovenia presented the situation in the construction sector. The goals of the discussion were to gain feedback on the usefulness of the Heat-Shield platform, to set a good example on heat stress mitigation, to find out more about negative heat impacts, especially for outdoor workers, and to discuss further options for cooperation and improved communication.

### 2.4. Fourth Step: Research on Employees’ Opinions on Heat Stress and Measures, Supplemented by Interviews with Employers

After gathering basic information about rising summer temperatures and general knowledge about heat stress, and sharing knowledge and ideas, it was important for us to get a more comprehensive picture of the situation of workers in many different companies. This was a supplement to our previous knowledge, which we had gained in 2016 in a production company [1], among agricultural workers and consultants [13] and among outdoor workers [14]. The questionnaire on heat stress among workers was designed by the Association of Free Trade Unions of Slovenia and distributed to its members by e-mail in June 2019. Responses were received from 209 workers, 60% of whom were men; 32% were under 40, 47% were between 41 and 55 and 21% were older. The workplaces were in many different sectors, 66% of which were indoors, 16% near hot equipment and the rest outdoors. The questions were mainly related to their working conditions when it is very hot and the measures taken by their employers.

During a heat wave in summer 2019, we tried to reach companies with potential heat problems to offer advice and get feedback from their management. We conducted phone interviews to gather the following information: location, sector, number of employees and where workers mainly work (indoor/outdoor, shade/sun). We also asked if there have been problems due to heat stress lately and what kind, was additional sick leave taken, which groups are more vulnerable, has productivity been reduced, has heat been a problem for workers in recent years, do you follow the weather forecast, do you measure temperature and humidity, what measures do you introduce in case of heat and when do you start (at what temperatures).

## 3. Results

### 3.1. Changed Distributions of Summer Temperatures and Increased Number of Meteoalarms Issued

**Figure 2:**
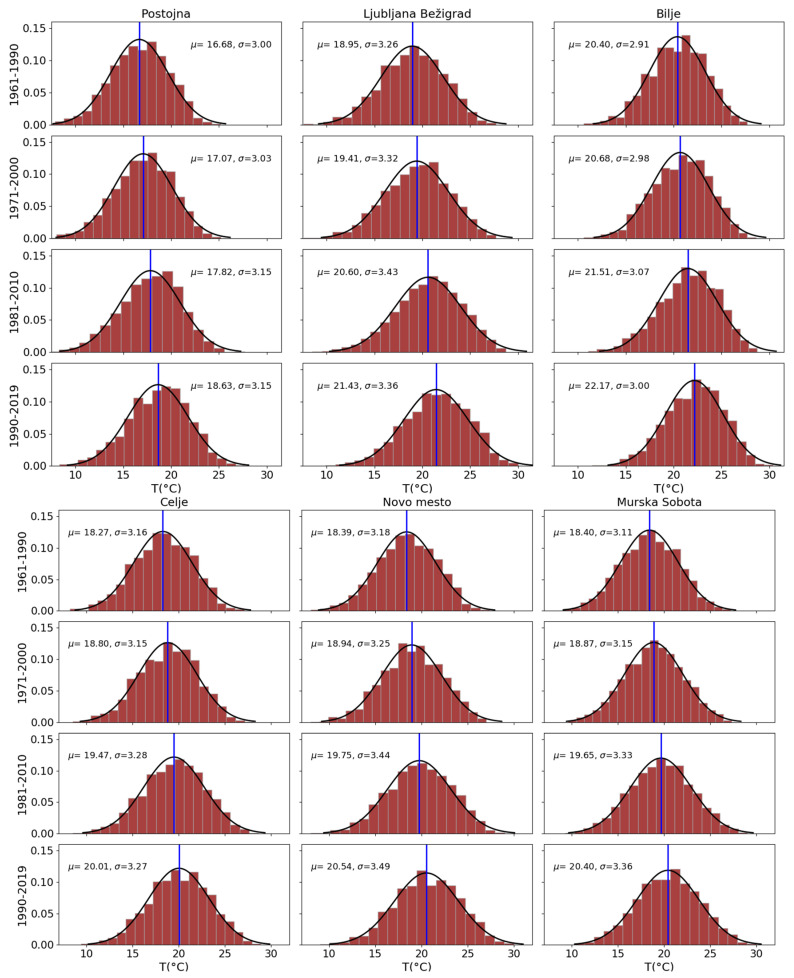
Distribution of summer (June–August) daily average temperatures in four periods (Bilje from 1962) in six locations in Slovenia.

**Figure 3:**
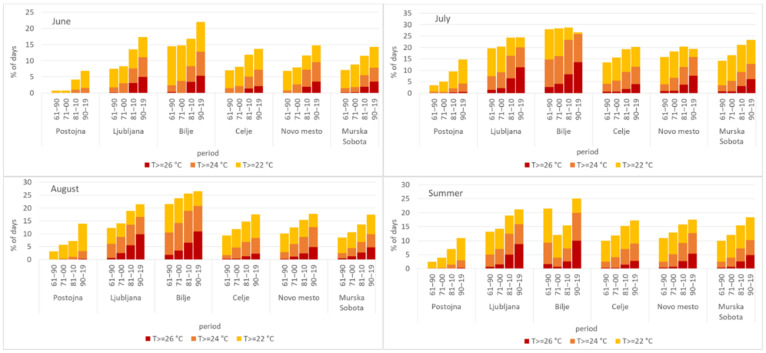
The percentage of days in summer (June–August) with average daily air temperature higher than or equal to the threshold (22 °C—yellow, 24 °C—orange and 26 °C—red) in four periods (1961–1990 (Bilje from 1962), 1971–2000, 1981–2010, 1990–2019) and six locations in Slovenia.

The six sites were selected because they represent different parts of Slovenia well, except for higher elevation sites, which are not so relevant from the point of view of heat stress. Thirty-year periods already represent the climate of a location, so we used them to represent climatological changes and not only interannual variations. The parameters of summer average daily temperature distributions have changed (Figure 2). The mean values (at the top of the curve) have shifted to the right by about 2–3 °C over time. In Postojna the shift was from 16.7 °C (1961–1990) to 18.6 °C (1990–2019); the temperatures there were the lowest. The temperature distributions in Celje, Novo mesto, and Murska Sobota, which increased from about 18 to over 20 °C, are very similar. Even higher are the temperatures in Ljubljana and Bilje, where they rose from 19 to 21.4 °C and from 20.4 to over 22 °C, respectively. In Novo mesto, it is most obvious that the distribution is becoming more flattened and the increase in the number of the hottest days is significant. To get a better impression, the proportions of the above three high temperature thresholds have been calculated for summer (June–August) (Figure 3). Average daily temperatures above 22 °C were rare in Postojna in the first two periods, and below 15% in all other locations except Bilje in the first period (21.4%). In the last period, all percentages were above 17% (except Postojna, 11%), and in Bilje up to 25%. Ljubljana had one day in five with temperatures above the threshold of 22 °C and Bilje had one day in four. The next threshold (24 °C) was almost never reached in Postojna before the last period, when there were only 3% of days with temperatures above that threshold. For Celje, Novo mesto and Murska Sobota, the percentage rose from about 2.5% to about 10%, in Ljubljana to 16% and in Bilje to 20%. During the period 1961–1990, average daily temperatures of 26 °C or above were very rare—below 1% of all days, except in Bilje (1.6%). In Postojna (0.3%), Celje (2.8%) and Murska Sobota (4.8%), the percentage remained below 5%; in Novo mesto it reached 5.4%, in Ljubljana 8.7% and in Bilje 10%. The highest proportion of days with high temperatures was always in July.

**Figure 4:**
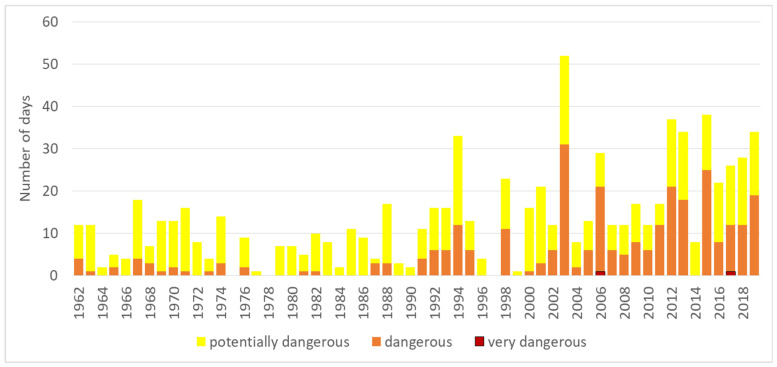
The number of days with fulfilled criteria for the potentially dangerous (yellow), dangerous (orange) and very dangerous (red) levels of meteoalarm in Bilje in the period 1962–2019.

The specific criteria that apply to meteoalarms were frequently met for the yellow (potentially dangerous) alarm, less frequently for the orange (dangerous) alarm (Appendix A) and very rarely for the red (very dangerous) alarm. The thresholds are different for each location (Table 1). The number of days with fulfilled yellow and especially orange meteoalarm conditions has increased significantly, with *p*-values for both linear trends lower than 0.001. In Bilje, 1996, 1997, 1999 and 2014 were the only years without orange alarms after 1990, in Ljubljana only 1996 and 1997 were without orange alarms after 1990, and in Murska Sobota 1991, 1995, 1996 and 1997. Most days with fulfilled conditions occurred in Bilje; there were only two days with red alarms in 2006 and in 2017, while there were more than 10 days with orange alarms in 1994, 1998, 2003 (maximum = 31 days), 2006, 2011–2013, 2015 and 2017–2019 (Figure 4). Red alarm conditions were met in Ljubljana one time each in 2003, 2012 and 2017 and five times in 2013; in Novo mesto they were met once in 2003, three times in 2013 and four times in 2019; in Murska Sobota they were met twice in 2007 and 2013 and once in 2017; they were met once in 2013 in Celje and never in Postojna. The years with the most numerous orange alarms at all locations were 2003, 2012, 2013, 2015 and 2017, mainly more than 10 days per year (except in Postojna, where the maximum was five times per year).

### 3.2. General Knowledge about Heat Stress and Warnings

**Figure 5:**
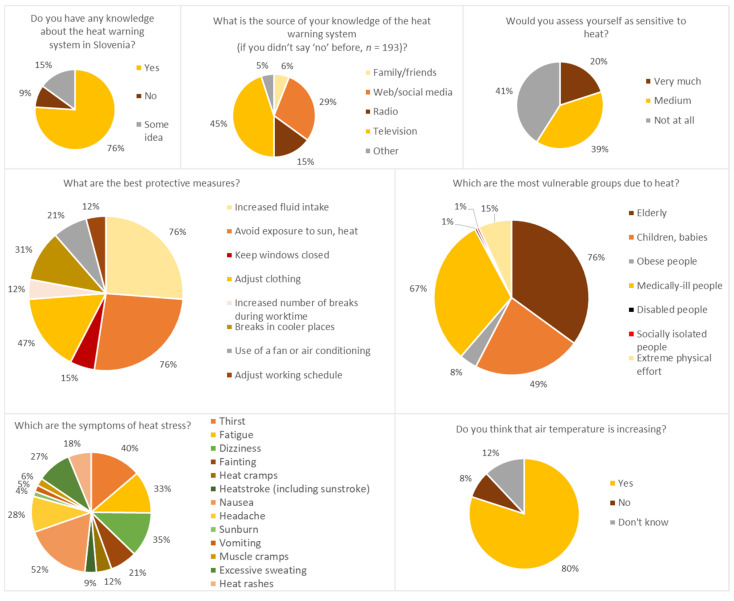
Response of the general public (*n* = 213) to questions about heat warnings and heat stress, collected in Ljubljana, Slovenia in the summer of 2017.

The survey of the general public (*n* = 213) in Ljubljana showed some interesting features (Figure 5). A large majority of the participants knew (76%) or had at least heard of (15%) the heat warning system that exists in Slovenia as a meteoalarm. There were no big differences between respondents in terms of age group, gender or education level. However, we found it surprising that, despite the large amount of information available nowadays through the web and social networks, only 29% of the participants received information online, while the vast majority of them received information through the television (45%). There were some differences here: People that are more educated chose the web more often than the less educated, and older people more often chose television as a source of information. The best-known risk groups due to heat were elderly people (76%), people with medical conditions (67%) and children/babies (49%). When people thought about reducing or mitigating heat stress, they mainly said that they avoided heat sources (including sunlight), increased their fluid intake (both: 76%) and adjusted their clothing (47%). Nausea (52%), thirst (40%), dizziness (35%) and fatigue (33%) were the most common symptoms of heat stress. Younger age groups stated thirst more often than older ones. The majority (80%) of the participants confirmed that, in their opinion, the temperature in Slovenia was rising, and a high percentage (41%) of them stated that they were not sensitive to heat at all.

### 3.3. Summary and Concise Ideas from the Two Stakeholder Symposia on Heat Stress

The symposia were organized within the Heat-Shield project [27], with the aim of raising awareness, developing plans to mitigate the negative effects of heat stress and taking a first step towards the broad use of the Heat-Shield platform, which was developed within the project as an early warning system for occupational heat stress [28]. The main outcomes of the first symposium’s roundtable discussions were:

- Employers: Motivation to work together is necessary, awareness is low, management teams do not realize that they are losing money due to poor working conditions; employers need to know that remedial action is not an extra cost but the only way to maintain productivity in the heat. In many sectors such as construction, exposure to sun and heat is still not recognized as dangerous. Where cooling with systems such as air conditioning is not possible, it is important to implement personal cooling strategies (e.g., cooling vests).
- Legislation: There is no explicit requirement of what an employer should do if the temperatures in the workplace become too high. Nor is the temperature threshold specified—except for 28 °C, when a cooler room must be made available for indoor workers. However, there are currently no legal restrictions for outdoor workers regarding maximum working temperatures. Some laws need to be revised: for example, higher trees (which would provide shade) around buildings in the industrial zone are not allowed.
- Warning systems: There are many ways to improve: For example, large screens in cities with recommendations to drink more and look for shade; the ARSO forecast and meteoalarm have recently become more user-friendly (also on Facebook, Twitter), but radio is still an important medium for elderly people and drivers.
- Media: Journalists need to find new alternatives for sharing information about the severity of the negative effects of heat. We encourage them to use examples of good practice, such as increased productivity using various methods to alleviate heat stress, which at the same time leads to fewer health problems and more money remaining in the public health fund.
- Education: Awareness of the negative health effects of heat waves/sunshine must be passed on to the education system so that people realize that they need to protect themselves and their health. Targeted, continuous information campaigns on the health of workers and the necessary measures to mitigate the effects of heat waves (and solar radiation) are needed for adults. The involvement of the media, businesses and trade unions is essential. Misunderstandings still exist, for example, claiming that sweating is not good.

The outcomes of the second symposium were the following: The right time, the right channel and the right level of communication are very important, and meteorologists are not properly trained to select them. In order to achieve positive changes, cooperation between different sectors is essential. The Traffic Safety Agency of the Republic of Slovenia will try to ensure that the negative effects of heat and appropriate reactions are included in the training. The Heat-Shield platform is a very useful heat health warning system for employers and employees, providing more specific short- and long-term forecasts with proposed measures to mitigate the negative effects of heat stress. A good example of heat stress mitigation from the production company “odelo” is that all workers have access to water at a distance of no more than 6 m, and ice cream, apples and isotonic drinks are available once a week. Additionally, the company begins to prepare for the heat in spring, when the cooling systems are cleared after the trees have blossomed and measures are taken as soon as the morning outdoor air temperature rises above 18 °C. In the new building, the air is exchanged five times per hour (in the old building it was only twice). On the other hand, construction workers consider the heat measures taken by their employers to be insufficient and pointed out that employers are saving on materials for protective clothing. Some workers would like to get a subsidy for working in the heat, but the position of the trade union is not to deal with health, but to address the heat problem. For example, mobile cooling units could be used. Postponing working hours can be problematic as people often travel a long way to work. During the heat, sleep is compromised anyway, and if they get up two hours earlier, this could have a negative effect on their health. Another problem is the social status of workers, because even if a minimum standard of living is set, this does not mean that workers can cool off at home or get a good night’s rest.

The main outcomes of the two symposia comprise the following: comprehensive presentations of heat climatology will be further used for educational purposes and to raise public awareness via traditional media, moreover several institutions agreed on collaborating in order to address heat stress issues more effectively. In addition, a collaboration with the Trade Union was established. Several companies started to use the Heat-Shield platform and got involved in the last year of the project to test the effectiveness of the heat mitigation measures.

### 3.4. Evaluation of Working Conditions at the Workplace by Workers during the Summer and Employers’ Views on the Heat Problem

After the exchange of knowledge among the stakeholders at the two symposia, it was important for us to use the summer of 2019 to obtain some additional information about the occupational setting during the hot months. The questionnaire, administered to 209 employees, had some unexpected results: According to the employees, the temperature at their workplace during summer can be up to 25 °C for 9% of the participants, up to 28 °C for another 15%, up to 30 °C for 21%, up to 35 °C for 27%, up to 40 °C for 18% and even above 40 °C for 10%. Under these very hot working conditions, employers mainly used air conditioning (inside) for cooling (56%), free drinks (24%), more frequent breaks (18%) and a cooler place to retreat from the heat (16%). However, 16% of employers did not use any of these remedial measures, and only 2% of employers interrupted work in extremely high temperatures. As a result, workers felt less productive (54%), had heat-stress symptoms such as exhaustion, headaches, nausea, etc. (54%), could not maintain their concentration (34%), were dehydrated (26%) and even fainted (21%). Therefore, 73% of the respondents said that the employers were not doing enough, and 45% of them said that the employers were not listening to the employees’ representatives. Employees mainly suggested using air conditioning wherever possible, and otherwise ensuring good ventilation, as well as more breaks and opportunities to drink. There should be legislation on what the temperature in the workplace should be, how many breaks there should be, the availability of drinking water and a cooler room and materials for summer clothing, with effective monitoring.

During that same summer, we contacted management representatives in several companies (not connected to employees above), but they were not very interested in the issue and claimed they were too busy. Only seven out of 60 were willing to participate in the survey. In three companies, the employees have the opportunity to use air conditioning. In only two companies do managers see heat stress as a health and economic problem, as workers sweat a lot, are tired and their productivity is decreased. With one exception, employers claimed that heat has become a real problem in recent years due to more frequent heat waves, higher temperatures and climate change in general. Managers (or CEOs) monitor the weather forecast for one (1×), three (3×) or more (3×) days in advance; in three companies, they measure the temperature and relative humidity. When temperatures rise to around 30 °C, five of them start taking some action: providing drinking water, previously cooled busses, parking in the shade and air conditioning, changing working hours and offering more frequent breaks with drinking water.

## 4. Discussion

In recent years, we have tried to create a solid basis for better heat communication and a heat action plan. Communication about rising summer temperatures with a focus on heat stress must have several levels. If we want to show the different stakeholders the seriousness of the problem, it is not enough to show them trends. Even though many people are aware of global warming, it is often noticed that they do not relate it to their own situation. That is why we are always looking for a way to show stakeholders how summer temperatures are changing in Slovenia [14]. This includes our results on changing distributions, especially with calculated percentages of exceedance of the threshold values. Similar approaches were followed in Florida by Raghavendra et al. [29], where it was shown that the shift in the mean temperature may increase the intensity of future heatwaves by 3–4 °C, while a flattening of the temperature distribution may enhance the intensity by an additional 1–2 °C The research on general knowledge showed that a large proportion of the participants (80%) think that temperatures in Slovenia will rise. In Madrid, 13% of respondents observed no increase in air temperatures, and in Lisbon that was as high as 23% [26].

The only system we have in Slovenia is the meteoalarm of the Meteorological Office (ARSO), which gives warnings at different levels. On the web, a warning is visible on the first page only for the current and next day; more information (for the next five days) is available when you “click” on it. In high heat, warnings are also published on the web and social media by National Institute of Public Health. A similar system is in use in the UK, for example, where recommendations for protections against heat are published by the National Health Service, Public Health England and the Met Office [30]. However, in France in 2006 it was confirmed that significant mortality was still observed during the heat wave, even where the observed temperatures remained below the heat warning thresholds [31], which means that efforts to promote prevention must continue, with increased communication of heat-related risks and appropriate behavior throughout the summer [32]. In addition, there was very rarely a case of a red (very dangerous) meteoalarm in Slovenia and there is a possibility that people will not take the threat seriously enough until the alarm reaches its highest level. This is why we are aiming for the Heat Health Action Plan, which is designed as a system with clear lines of responsibility for the different authorities involved [19], starting with the analysis of the effects of heat, bringing together different stakeholders, possible chain links and policy makers, and including employees as an overlooked vulnerable group. It is important to bear in mind that, for example, for workers carrying out roofing, road construction or interior work on a building without air conditioning and with poor ventilation, the temperature may be even higher than the outside air temperature [33].

Our analyses and research on general knowledge gave us an idea of the current situation in Slovenia in order to compare it with some other countries. For example, a national survey found that 55% of participants had heard of heat protection recommendations during the 2013 heat wave in the UK [30], while in Slovenia only 9% had never heard of heat warnings. Questions in the survey were from the questionnaire used in Brussels (among 120 passers-by) and Amsterdam (133) in summer 2015 [25], and in summer 2016 in Lisbon (129 passers-by) and Madrid (131) [26]. An important piece of information in Slovenia was that older people reported less thirst as a heat stress symptom than younger people, and also less often reported drinking more fluids. Younger people also tend to use a fan or air conditioning more. Increased fluid intake was also mentioned more often by women than by men, which was confirmed at our meeting with stakeholders using an example from the manufacturing factory. In Brussels and Amsterdam [25], respondents also indicated that they mainly reported increased fluid intake (81% and 59%, respectively) for the prevention of heat stress, with similar proportions in Lisbon and Madrid [26] of 74% and 73%, respectively. In none of the five cities mentioned did the participants talk about caring for those at risk. However, in another survey in France, the heat prevention plan has changed the awareness of heat-related risks in the general population, and 73% of the 1240 adult participants had taken measures to protect their elderly relatives and friends, including regular visits (39%) and regular telephone conversations (29%) during the 2006 heat wave [32].

In Lisbon, 36% of the participants considered themselves heat-sensitive, while in Madrid the proportion was 33% [26]. Given the lower summer temperatures in Brussels and Amsterdam, it is interesting that only 32% and 12% of the participants, respectively, consider themselves very heat-sensitive [25]; in Slovenia this was 20%. Respondents in Slovenia in the under 50 age group were more likely to consider older people to be heat-sensitive, while older people were more likely to report being medically ill. Of the older respondents in Brussels and Amsterdam, more than 20% were aware that older people are a risk group for heat, but did not consider themselves to be heat-sensitive, which could indicate that even with a good level of individual knowledge, one’s own risk can be misjudged [25]. In the UK, it was found that heat protection recommendations can undermine their implementation by producing positive affect about hot weather [30].

As in some other countries [26], the message to be sent to stakeholders was that efforts to raise awareness of heat must be stepped up and that public health measures should effectively target vulnerable groups. The stakeholders’ symposium provided an important discussion on different effective ways to address the most vulnerable groups (including workers) and the people who care for them. Agencies (ARSO), institutes (NIJZ), policy makers and the media need to play an active role in this process. Very little information is available for older people on television and radio, and much more on the web and social media, which they do not use to this extent. In many respects, it became clear that further education and public relations work is important, especially with regard to the simple and understandable presentation of trends and climate projections. One example of good practice is the Slovenian educational TV show *Bite into Science* on heat, the idea for which came from the first symposium. Education and communication strategies are very important to raise awareness of the danger so that the population is prepared for a heat wave [9].

Stakeholder symposia and further research have shown that the level of awareness or understanding of the negative effects of heat stress on workers’ health and productivity [18] is still quite low, so we need effective ways of exchanging information between different economic sectors. Prevention and mitigation plans that lay out step by step what needs to be done at an individual or company level at each stage of the occurrence of heat stress would be a great help. In some industrial environments, the cooling of large production halls [34] is not feasible or, in the opinion of management, too costly. In such cases, personal cooling strategies should be presented, and the focus should be on regulating the thermal conditions in the immediate vicinity of the workplaces. Cooling jackets are one of the ways to promote evaporation and cool down at work [35,36,37]. The Heat-Shield platform feels useful, transparent and helpful for the participants of both symposia. A strength of the developed website platform lies in its ability to make short-term recommendations on hydration and work and break schedules [28]. Most stakeholders agree that it is easiest and most efficient to introduce preplanned breaks during working time, but it must be clearly stated that this measure does not reduce workers’ productivity [38], which is a major concern of employers. Both researchers and stakeholders recognize the importance of chilled rooms (or, if outside, at least shading and ventilation) and cold drinking water. Sufficient hydration is also of great importance. Although many stakeholders are aware of this, research in Denmark has shown that about 70% of employees come to work dehydrated [39], which undoubtedly affects their productivity. Meanwhile, employers in some sectors discourage employees from drinking so as not to waste time on frequent visits to the toilet. The last two studies also found that workers already have heat-induced problems, but the majority of employers are not yet prepared to deal with the negative effects of heat stress, similar to the situation in Europe [40]. Knowledge, trust, willingness to act and implementation possibilities vary greatly from company to company. We; therefore, believe that information on more effective but costly measures to reduce heat stress should be offered to those who are most interested or most exposed, and the focus remains on strategies that strike the best balance between efficiency, feasibility and economic cost. For example, work-shifting, working in the shade [41] and the abovementioned measures. The money-making impulse is so strong that the implementation of many measures is largely possible only through new legislation. Recommended measures that can be taken in advance include increasing shade from outside the building by, for example, planting higher trees [42], which is not allowed in industrial zones in Slovenia. The trade unions are trying to prepare new legislation on working in hot weather, with defined temperature thresholds for outdoor workers and urgent measures, following the recommendations [43,44] and discussed results of both symposia.

We believe that the limitations of our case studies will be overridden by the benefits that all parties gain in working towards a better heat warning system. Our results are not necessarily representative of the whole country but give a good indication of the knowledge and perception of heat stress in Slovenia and the warning system we already have.

## 5. Conclusions

The present study has given insight into the development process of a Slovenian heat health warning system. The parameters of the summer average daily temperature distributions have changed in Slovenia in the period 1961–2019: The mean values have shifted to the right by about 2–3 °C over time, and the number of days with fulfilled criteria for dangerous and very dangerous heat-related conditions has increased, especially since 1990. Stakeholder symposia and further research have shown that the level of awareness of the negative effects of heat stress on health and productivity is still quite low. Workers have heat-induced problems, but the majority of employers are not yet prepared to take action against heat stress in the workplace. There are also currently no legal restrictions for outdoor workers with regard to maximum working temperatures.

Prevention and mitigation plans are needed to alleviate heat stress in the very near future. Awareness of the negative health effects of heat waves must be passed on to the education system through targeted, continuous information campaigns on workers’ health, and necessary measures to mitigate the effects of heat stress are needed. Platforms for announcing daily heat stress risk levels and behavioral suggestions (hydration, work breaks) should be available to issue short- and long-term heat risk forecasts for planning outdoor and workplace activities. New legislation on hot-weather work, with defined temperature thresholds for outdoor workers and accompanying measures, is urgently needed. The involvement of the media, companies and trade unions in all of the above activities is essential. An improved heat warning system will lead to fewer health problems and a more financially efficient public health fund.

## Figures and Tables

**Table 1:** Meteoalarm warning level criteria for heat in Slovenia (T_max_—maximum daily temperature, T_avg_—average daily temperature) [24].

	Region				
Meteoalarm Warning Level	Southwestern	Northwestern	Central	Northeastern	Southeastern
None					
Potentially dangerous	T_max_ > 32 °C	T_max_ > 29 °C	T_max_ > 31 °C	T_max_ > 31 °C	T_max_ > 32 °C
Dangerous	T_max_ > 35 °C and/or T_avg_ > 26 °C	T_max_ > 33 °C and/or T_avg_ > 23 °C	T_max_ > 34 °C and/or T_avg_ > 26 °C	T_max_ > 34 °C and/or T_avg_ > 26 °C	T_max_ > 35 °C and/or T_avg_ > 26 °C
Very dangerous	T_max_ > 38 °C and T_avg_ > 28 °C	T_max_ > 35 °C and T_avg_ > 26 °C	T_max_ > 37 °C and T_avg_ > 28 °C	T_max_ > 37 °C and T_avg_ > 28 °C	T_max_ > 38 °C and T_avg_ > 28 °C

## Appendix A

**Table A1:** The number of days in each summer from 1961 (in Bilje from 1962) to 2019 with fulfilled conditions for the yellow (first column, potentially dangerous), orange (second column, dangerous) or red (third column, very dangerous) meteoalarm at six locations in Slovenia.

Year	Postojna			Ljubljana			Bilje			Celje			Novo Mestos			Murska Sobota		
1961	0	0	0	7	0	0	−	−	−	4	0	0	3	0	0	5	1	0
1962	0	0	0	9	0	0	12	4	0	7	0	0	6	0	0	3	0	0
1963	0	0	0	6	0	0	12	1	0	10	1	0	3	1	0	8	1	0
1964	0	0	0	1	0	0	2	0	0	1	0	0	1	0	0	1	0	0
1965	1	0	0	6	1	0	5	2	0	6	1	0	2	0	0	1	0	0
1966	0	0	0	1	0	0	4	0	0	0	0	0	0	0	0	0	0	0
1967	0	0	0	8	1	0	18	4	0	10	0	0	4	0	0	4	0	0
1968	2	0	0	5	3	0	7	3	0	4	2	0	3	2	0	4	3	0
1969	1	0	0	2	0	0	13	1	0	2	0	0	0	0	0	2	0	0
1970	0	0	0	4	0	0	13	2	0	4	0	0	2	0	0	1	0	0
1971	2	0	0	11	2	0	16	1	0	5	1	0	1	1	0	5	1	0
1972	1	0	0	4	1	0	8	0	0	3	0	0	1	0	0	1	0	0
1973	0	0	0	1	0	0	4	1	0	0	0	0	0	0	0	0	0	0
1974	3	0	0	8	0	0	14	3	0	4	0	0	1	0	0	4	0	0
1975	0	0	0	2	0	0	0	0	0	1	0	0	0	0	0	0	0	0
1976	0	0	0	6	0	0	9	2	0	8	1	0	2	0	0	4	0	0
1977	0	0	0	1	0	0	1	0	0	4	0	0	0	0	0	1	0	0
1978	0	0	0	0	0	0	0	0	0	0	0	0	0	0	0	0	0	0
1979	0	0	0	2	0	0	7	0	0	3	0	0	1	0	0	0	0	0
1980	0	0	0	4	0	0	7	0	0	2	0	0	0	0	0	0	0	0
1981	0	0	0	4	0	0	5	1	0	4	0	0	0	0	0	2	0	0
1982	0	0	0	5	0	0	10	1	0	3	0	0	0	0	0	1	0	0
1983	5	0	0	13	6	0	8	0	0	9	4	0	6	4	0	6	1	0
1984	0	0	0	2	1	0	2	0	0	3	1	0	2	0	0	1	0	0
1985	0	0	0	7	1	0	11	0	0	3	0	0	1	0	0	4	1	0
1986	0	0	0	9	0	0	9	0	0	7	0	0	2	0	0	4	0	0
1987	0	0	0	4	0	0	4	3	0	4	1	0	0	1	0	4	2	0
1988	4	0	0	20	4	0	17	3	0	14	0	0	11	1	0	17	4	0
1989	0	0	0	1	0	0	3	0	0	1	0	0	0	0	0	0	0	0
1990	0	0	0	5	0	0	2	0	0	7	0	0	3	0	0	5	0	0
1991	1	0	0	6	2	0	11	4	0	3	0	0	2	0	0	5	0	0
1992	9	2	0	22	8	0	16	6	0	21	10	0	16	3	0	27	16	0
1993	6	0	0	16	2	0	16	6	0	16	3	0	12	4	0	19	6	0
1994	4	0	0	23	6	0	33	12	0	19	1	0	9	3	0	22	1	0
1995	1	0	0	8	1	0	13	6	0	4	0	0	0	0	0	2	0	0
1996	0	0	0	5	0	0	4	0	0	4	0	0	0	0	0	4	0	0
1997	0	0	0	0	0	0	0	0	0	0	0	0	0	0	0	0	0	0
1998	9	0	0	22	6	0	23	11	0	13	2	0	5	1	0	14	3	0
1999	0	0	0	6	2	0	1	0	0	6	0	0	3	0	0	6	1	0
2000	7	0	0	16	4	0	16	1	0	18	5	0	15	7	0	24	7	0
2001	2	0	0	20	6	0	21	3	0	14	2	0	8	3	0	19	4	0
2002	1	0	0	9	4	0	12	6	0	7	2	0	4	1	0	9	2	0
2003	23	2	0	43	22	1	52	31	0	47	20	0	37	17	1	45	18	0
2004	1	0	0	8	2	0	8	2	0	8	1	0	2	1	0	6	1	0
2005	4	1	0	10	3	0	13	6	0	6	2	0	3	3	0	6	2	0
2006	9	2	0	23	9	0	29	21	1	17	5	0	10	8	0	18	2	0
2007	7	1	0	15	8	0	12	6	0	16	6	0	11	7	0	18	10	2
2008	1	0	0	5	3	0	12	5	0	7	2	0	4	0	0	8	1	0
2009	5	0	0	15	1	0	17	8	0	11	1	0	6	2	0	10	2	0
2010	3	0	0	15	9	0	12	6	0	13	6	0	11	6	0	16	8	0
2011	14	1	0	16	10	0	17	12	0	16	6	0	13	6	0	12	6	0
2012	21	4	0	32	19	1	37	21	0	32	10	0	23	16	0	32	17	0
2013	16	5	0	26	16	5	34	18	0	25	13	1	20	16	3	28	15	2
2014	2	0	0	9	2	0	8	0	0	8	1	0	4	0	0	9	2	0
2015	19	3	0	31	20	0	38	25	0	29	9	0	19	11	0	30	13	0
2016	2	0	0	17	4	0	22	8	0	7	0	0	4	3	0	9	3	0
2017	12	5	0	28	17	1	26	12	1	28	9	0	29	14	0	28	9	1
2018	9	0	0	22	7	0	28	12	0	19	0	0	4	0	0	21	2	0
2019	13	2	0	21	10	0	34	19	0	23	3	0	11	5	4	23	3	0
